# From Sir Henry Burdett, to the Editor of "The Times."

**Published:** 1919-06-14

**Authors:** Henry C. Burdett

**Affiliations:** The Lodge, 13 Porchester Square, W. 2, June 6.


### From Sir Henry Burdett, to the Editor of "The Times."

SIR,—My letter in the *Times* of 3rd instant stands in its entirety. It drew aside the veil and offered the solution of the mystery attaching to the actual number of individual nurses who are claimed as members of the societies with high-sounding names affiliated to the Central Committee.

Mr. Herbert Paterson's letter in the *Times* of to-day confirms the looseness of the figures laid before Standing Committee E. Mr. Paterson has "no doubt that the various societies" he represents "will be quite prepared to submit their evidence to the proper authorities." Justice to Parliament, the nursing profession, and the public requires in detail the actual number of fully-trained nurses who are supporters of the Central Committee and the evidence of their full training in each case. Will Mr. Paterson therefore, without further delay or argument, arrange for the various societies to submit their evidence to a Select Committee appointed by Parliament, with power to call witnesses, and so bring the actual facts to the test of demonstrable truth?

Mr. Macmaster, the Chairman of Standing Committee E, is reported to have suggested that the Central Committee's Bill should be referred back to his Committee, for the Committee had been misled, and those with whom he had discussed the subject agreed with him that the Bill as it stands is not right. There should therefore be neither delay nor difficulty in Mr. Paterson's path to arrange for a full inquiry to be made by the proper authorities.

The figures and Register submitted by the College of Nursing are open to inspection, and their accuracy is demonstrably provable.—I am, yours faithfully,

HENRY C. BURDETT.

The Lodge, 13 Porchester Square, W. 2, June 6.

(*The Times*, June 9.)

